# Retraction Note: miR-381 modulates human bone mesenchymal stromal cells (BMSCs) osteogenesis via suppressing Wnt signaling pathway during atrophic nonunion development

**DOI:** 10.1038/s41419-024-06650-5

**Published:** 2024-04-19

**Authors:** Haitao Long, Yong Zhu, Zhangyuan Lin, Jun Wan, Liang Cheng, Min Zeng, Yifu Tang, Ruibo Zhao

**Affiliations:** Department of Orthopaedics, Xiangya Hospital, Central South University, Changsha, 410008 China

Retraction to: *Cell Death and Disease* 10.1038/s41419-019-1693-z, published online 17 June 2019

The Editor’s in Chief have retracted this article. After publication overlap between images was noted in multiple figures, specifically:

- In Fig. 3H the Alizarin Red Staining NC mimics overlaps with the Alizarin Red Staining control in Fig. 5I.
- In Fig. 3H the Alizarin Red Staining miR-381 mimics overlaps with the Alizarin Red Staining NC inhibitor si-FZD3 in Fig. 5I.
- In Fig. 3H the ALP miR-381 inhibitor overlaps with the ALP NC inhibitor si-FZD3 in Fig. 5I.
- In Fig. 3H the ALP miR-381 mimics overlaps with the ALP control in Fig. 5I.
- In Fig. 3H the ALP NC mimics overlaps with the ALP PBS in Fig. 6K.
- In Fig. 5I the ALP miR-381 inhibitor +si-NC overlaps with the ALP PBS in Fig. 6K.

The Editor’s in Chief have, therefore, lost confidence in the integrity of the results presented in this article.

None of the authors have responded to any correspondence from the editor/publisher about this retraction.

